# The Rate of Cortisol Decline After Consuming a High-Fat Test Meal for Breakfast Partially Explained Sex-Dependent Variation in Post Ingestive Cardiovascular Status

**DOI:** 10.1016/j.cdnut.2026.107655

**Published:** 2026-02-06

**Authors:** Kevin D Laugero, Ryan G Snodgrass, Nancy L Keim

**Affiliations:** 1 Obesity and Metabolism Research Unit, USDA-Agricultural Research Service, Western Human Nutrition Research Center, Davis, CA, United States; 2 Department of Nutrition, University of California, Davis, Davis, CA, United States; 3 Diet, Microbiome, and Immunity Research Unit, USDA-Agricultural Research Service, Western Human Nutrition Research Center, Davis, CA, United States

**Keywords:** sex, postprandial, fasting, meal challenge, cortisol, sympathetic nervous system, cardiovascular function

## Abstract

**Background:**

Circulating cortisol concentrations are connected to diet and the neurological systems that regulate food intake. Skipping breakfast can diminish the typical drop in circulating cortisol concentrations.

**Objectives:**

Determine *1*) whether overnight fasted males and females differ in salivary cortisol (CORT) dynamics after consuming a high-fat test meal, *2*) if these differences in postprandial (PP) CORT explain variation in cardiovascular status later in the day, and *3*) whether PP CORT mediates sex-based differences in cardiovascular status.

**Methods:**

This cross-sectional study examined >300 male and female participants. The recruitment sampling scheme consisted of sex (male and female) × age (18‒33 y, 34‒49 y, 50‒65 y) × BMI (normal weight: 18.5‒24.99, overweight: 25‒29.99, and obese: 30‒39.99). The study test visit was conducted at the USDA Western Human Nutrition Research Center and included a high-fat test meal, an emotion-induction (anger recall) task, and collection of blood, saliva, self-reported mood, and physiological information under fasted, PP, and post anger-recall conditions.

**Results:**

After an overnight fast and following the test meal, PP CORT descended significantly (*P*_time × sex_ = 0.00008) more sharply in females compared with males. Fasting CORT did not differ (*P* = 0.620) between males and females, but females displayed lower CORT at 30 min (*P* < 0.007), 60 min (*P* < 0.005), and 90 min (*P* < 0.010) following the test meal. Higher PP CORT associated with elevated anger-induced heart rate (β: 0.050 ± 0.022; *P* = 0.0232), elevated anger-induced sympathetic tone (β: 0.232 ± 0.105; *P* = 0.0282), and lower endothelial function (β: ‒0.1637 ± 0.0698; *P* = 0.0197). Endothelial function, and anger-induced heart rate and sympathetic tone differed between males and females, with variation in PP CORT partially explaining these sex-based differences.

**Conclusions:**

The magnitude of CORT decline after consuming the first daily meal may contribute to sex-based differences in downstream cardiovascular reactivity.

## Introduction

Cortisol regulation is connected to diet and the neurological systems that regulate food intake. Increases in glucocorticoid concentrations that occur during a typical overnight fast stimulate the drive to eat and increase the magnitude of energy consumed at breakfast or the first meal of the day. Skipping breakfast leads to persistently higher circulating concentrations of corticosterone in rodents and cortisol in humans (e.g., [1,2]). Even modest elevations in this steroid hormone over the course of the day can have detrimental effects on cardiometabolic function [[3], [4], [5], [6], [7]]. In rodents, preventing consumption of the first meal of the day (lights off) significantly elevates corticosterone, which resolves when the animals are allowed to eat [1]. Increases in corticosterone also associate with food deprivation, and refeeding with either rodent unpurified diet [8,9] or an artificially sweetened drink [9] lowered plasma corticosterone concentrations within 1 h of refeeding. However, compared with the unpurified diet meal, the noncaloric drink was not as effective in reducing concentrations of corticosterone [9], highlighting the likely importance of a post ingestive and metabolic-related signal in cortisol regulation.

We previously showed in females that, compared with breakfast eaters, breakfast skippers had elevated salivary cortisol (CORT) concentrations throughout the morning and afternoon, due in part to higher cortisol concentrations preceding the first meal of the day [2]. Results from that study also supported the known circadian-based decline in circulating cortisol concentrations from waking to the first meal of the day in both groups. Overall, the study in humans supports a combination of circadian and dietary-based mechanisms that determine the magnitude of cortisol decline after waking and following the first meal of the day.

During times of overnight or prolonged fasts, when insulin concentrations are relatively low, cortisol elevation (or corticosterone in rodents) promotes glycogenolysis, gluconeogenesis, and lipolysis to support energy metabolism. At the same time, these steroid hormones act on the brain to promote the drive for food [10]. In combination with the typical circadian drive, consuming the first meal of the day and refeeding after a prolonged fast are associated with reduced activity in the hypothalamic-pituitary-adrenal (HPA) axis and a decline in circulating glucocorticoid concentrations. Together, the same neuronal systems that sense the ongoing status of energy metabolism project to brain pathways that regulate activity in the adrenal-cortical system [11]. This is of interest because cortisol dysregulation is central to both metabolic and psychiatric diseases, and its regulation may play a key role in biologically linking metabolic and psychiatric disease comorbidity [12].

Nutritional regulation of the HPA axis has been demonstrated under numerous contexts (e.g., [[13], [14], [15]]), and the stimulatory effects of prolonged fasting on basal activity in the HPA axis are well documented (e.g., [16,17]). However, much less is known in humans about variation in the cortisol response to the first meal of the day, and whether this variability explains differences in downstream health status or function. Although there exist several reports describing cortisol induction after a midday meal or sex-related differences in HPA axis regulation (see [18]), less is known about the influence of consuming a mixed macronutrient meal in the morning on cortisol concentration dynamics. Moreover, whether this early meal-cortisol response relationship varies between males and females is not well described. Therefore, we tested whether males and females differ in the cortisol response to consuming the first meal of the day, and whether this sex-related variance in the postprandial (PP) cortisol response explained differences in cardiovascular reactivity and emotion-affiliated mood and autonomic nervous system reactivity later in the afternoon. This paper represents a secondary examination of outcomes. Primary outcomes/hypotheses are detailed in Baldiviez et al. [19].

## Methods

### Subjects

Research participants were from the nutritional phenotyping study (phenotyping study; clinicaltrials.gov: NCT02367287), which was conducted at the USDA Western Human Nutrition Research Center (WHNRC) in Davis, CA. The phenotyping study [19] is a cross-sectional study that recruited 393 generally healthy males and females living near Davis, CA. The determination of sample size was based on the primary outcomes in the cross-sectional study and detailed in [19]. Males and females with BMI between 18.5 kg/m^2^ and 40 kg/m^2^ and between 18 y and 65 y old were recruited into the study. As previously described [19], efforts were made to enroll participants across equal distributions of 3 age bins and 3 BMI bins by sex. The recruitment sampling scheme consisted of sex (2 levels consisting of male and female) × age (3 levels consisting of 18‒33 y, 34‒49 y, and 50‒65 y) × BMI (3 levels consisting of normal weight: 18.5‒24.99, overweight: 25‒29.99, and obese: 30‒39.99). Subjects came to the WHNRC for 2 test visits, which occurred within a period of 6–55 d apart, with a mean of 14 d. This report focuses solely on the second test visit, which included both a meal challenge and an anger-recall task; see below. The study was approved by the University of California, Davis institutional review board. All participants provided written informed consent and received monetary compensation for their participation.

### Study design

The night before the test visit and before 19:00, participants were asked to eat a provided and relatively high-carbohydrate dinner consisting of 873 total kcals (∼17% kcal from fat, ∼74% kcal from carbohydrate, and ∼9% kcal from protein) [20], which was prepared by the WHNRC Metabolic Kitchen and Human Feeding Laboratory. After consuming this standardized meal, participants fasted for 12 h. Upon arrival the next morning, fasting saliva and blood were collected and then again at predetermined time intervals after the participant consumed a mixed macronutrient liquid challenge meal (60% kcal from fat, 25% kcal from carbohydrates, and 15% kcal from protein). Participants were also affixed with an electrode system on the palm of their non-dominant hand for examining skin conductance, which is a marker of sympathetic nervous system (SNS) activity. For CORT, saliva was collected immediately before the test meal and 30 min, 60 min, and 90 min after the test meal was consumed. At ∼2 h after the 90-min PP saliva sample (3.5 h after consumption of the test meal), an anger-recall task was conducted. The task developed for this study was based on that described by Moons and Shields [21]. The task was introduced by asking the participants to recall an unresolved conflict that is still a source of anger. After a 2-min private self-reflection of the conflict, the participants were asked to share details aloud to a trained study staff member over a 3-min period about their experience and feelings related to the recall of this conflict. CORT, skin conductance, and anger via the 65-item Profile of Mood State (POMS) questionnaire were collected before and after the task. We used the POMS to specifically query present-moment anger to verify the functional effectiveness of the anger-recall task. The anger-recall task lasted 5 min. In addition to collecting saliva in response to the test meal, saliva was also collected immediately before and 30 min, 60 min, and 90 min after the anger-recall task. Saliva was analyzed for cortisol concentrations.

**FIGURE 1:**
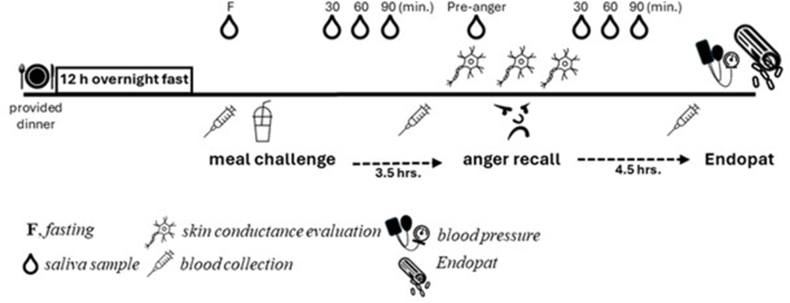
Schematic diagram represents the types and relative time of activities before and during the study test visit.

The challenge test meal was a modified version of the mixed macronutrient tolerance test meal used by Wopereis et al. [22]. The composition and liquid form were originally selected to more broadly and simultaneously examine in a non-diabetic population multiple metabolic and physiological responses related to insulin sensitivity, lipid tolerance, and metabolic flexibility [19]. This mixed macronutrient, high-fat liquid test meal was 403 g and ∼800 kcal, which consisted of ∼60% kcal from fat (palm oil), ∼25% kcal from carbohydrates (sucrose), and ∼15% kcal from protein (egg white). Participants were given 10 min to consume the test meal. The mixed macronutrient test meal was prepared by the Metabolic Kitchen and Human Feeding Laboratory. Approximately 4.5 h after the anger-recall task (8 h after consumption of the test meal), endothelial function (EF) was examined using an EndoPat device (Itamar Medical Ltd.), as previously described [19]. Refer to the schematic diagram (Figure 1) for the types and relative time of activities before and during the test visit. This diagram represents a subset of tasks relevant to this analysis.

### Measurements

#### Skin conductance and heart rate

A hardware and software (BioLab 3.4.1) system provided by MindWare Technologies Ltd. was used to collect skin conductance and heart rate (HR) data. To record skin conductance, electrodes were placed on the palm of the non-dominant hand. Generally, skin conductance levels or responses can be used to indicate emotional arousal and SNS activity [23,24]. Skin conductance responses (SCRs) reflect nonspecific or specific event-related transient increases in the skin conductance. The frequency of these responses can be used to detect task-related increases in arousal or SNS activity. In this study, we examined tonic (average skin conductance level measured in microSiemens (μS) excluding SCRs) and total SCR (task-related phasic increase; minimum change set to 0.05 μS threshold) before, during, and after the anger-recall task. Data were processed and cleaned for artifacts using electrodermal activity (EDA) software (EDA 3.1.0) from MindWare Technologies. Electrocardiogram data were also collected using MindWare BioLab 3.4 to estimate HR. Electrodes were placed on the body as predetermined by the manufacturer. A positive electrode was placed at the bottom left rib near the side, a negative electrode was placed on the right collar bone (clavicle), and the ground electrode was placed at the bottom right rib on the subject’s side. The mean HR in beats per minute was derived in the heart rate variability (HRV) output file using a software package (HRV 3.2.11) from MindWare Technologies. Data used to estimate HR were taken from a 5-min collection period immediately before, during, and after the anger task.

#### Salivary Cortisol

Saliva samples were collected using Salimetrics oral swabs. Research volunteers placed the swab in their mouth for 1–2 min and then deposited the swab into the provided sample tubes. Swab samples were immediately centrifuged to collect and aliquot saliva, and then stored at −80°C until being assayed for cortisol at the WHNRC. ELISA (expanded-range high-sensitivity salivary cortisol kit; Salimetrics) was used to determine CORT concentrations. This assay can detect cortisol concentrations ranging from 0.193 nmol/L to 82.77 nmol/L (0.007–3.0 μg/dL) and has a typical intra- and interassay coefficient of variation of 3.5% and 5.1%, respectively.

#### Vascular function

An EndoPat system (Itamar Medical Ltd.) was used to examine peripheral microvascular function, as previously described [25]. Reactive hyperemia index (RHI) was used to assess endothelial function (EF), with a higher RHI indicating better EF. Reactive hyperemia is an increase in blood flow following transient arterial occlusion, which is dependent on the ability of blood vessels to dilate properly. Immediately prior to the EndoPat procedure, blood pressure was measured using a standard blood pressure cuff placed on 1 arm.

#### Inflammatory marker measurements

Plasma concentrations of C-reactive protein (CRP), TNF-α, and IL-1β, IL-6, IL-8, and IL-10 were examined immediately before the test meal (0 h) and 3 h and 6 h after meal consumption. As previously described [26], MSD (MESO Scale Discovery) assay kits and the MSD sector imager 2400 were used to analyze EDTA plasma for these markers. CRP was analyzed using the MSD Vascular Injury Panel 1, and cytokines were analyzed using the Vplex Custom Human Biomarker Proinflammatory Panel 1.

#### Physical measurements

Subjects were weighed wearing lightweight surgical scrubs using a calibrated electronic scale (Tanita BWB-627A class III electronic scale; Toledo Scale) to the nearest 0.1 kg. A wall-mounted stadiometer (model S100; Ayrton Corporation) was used to measure height to the ∼0.1 cm. BMI was calculated.

### Statistical analysis

Secondary outcomes examined in this work included PP CORT, anger-affiliated HR and SNS activity, anger task-affiliated CORT, vascular function, blood pressure, and markers of inflammation. Of these, PP CORT, anger-affiliated HR and SNS activity, anger task-affiliated CORT, vascular function, and blood pressure were the primary focus of this secondary analysis. SAS for Windows, version 9.4, was used for all statistical analyses. We applied logarithmic transformations to the cortisol, HR, tonic skin conductance, SCR, CRP, IL-1β, IL-10, and reactive hyperemia data prior to analysis due to non-normal distribution. Base 10 log transformations were applied to the cortisol variable, whereas natural log transformations were applied to HR, tonic skin conductance, SCR, CRP, cytokines, and reactive hyperemia variables. For the same reason, a square root transformation was applied to IL-6 and IL-8, and a rank-order procedure (proc rank) was used to produce rank-ordered values for reported anger from the POMS questionnaire. In some cases, a constant was systematically added prior to logarithmic transformation to ensure all values were positive before transformation. For nonrepeated variables, various transformations such as logarithm or square root were applied, and the resulting transformed variable was assessed for normality using the Shapiro-Wilk statistic. For repeated measures variables, transformations were applied, and the residuals from a 2-factor analysis of variance (with subject and time as categorical factors were assessed for normality in the same way). For repeated measures models, we also tested for heterogeneity of variance across time, and there is no significant difference among the variances. To examine the effects of sex on log CORT, we first applied a linear mixed model procedure [Proc Mixed (SAS MIXED procedure)] to test for associations between sex and cortisol concentrations across the fasting and PP timepoints. The mixed model included fixed effects of sex and time of saliva collection (fasting and 30 min, 60 min, and 90 min PP), and a 2-factor interaction of sex and time of collection. It also included random effects of subject and subject by time to account for repeated measurements across the saliva samples. For analyses involving the primary focus of this paper, which was meal challenge-related cortisol, only participants with a complete CORT dataset for the meal challenge were included in the analyses. For analyses involving the anger-recall cortisol, only participants with a complete CORT dataset for both the meal challenge and anger recall were included in the analyses. Because participant recruitment was stratified by sex, age category (bin), and BMI category (bin), these variables were included as covariates in all models. For the repeated measurement data, a time by sex, time by BMI category, and time by age category terms were also included. Proc Mixed was also used to test for effects of sex on the 30-, 60-, and 90-min PP change in log CORT. Fasting (pre-meal) CORT was also included in the models, testing effects of sex on change in cortisol (delta cortisol). Regression plots were produced in Microsoft Excel (Microsoft 365 MSO) using covariate-adjusted values. In all cases, *P* ≤ 0.05 was considered to be statistically significant.

Based on the repeated measures results, 3 separate mixed models were then used to test *1*) for an effect of sex on average PP log CORT concentration, statistically controlling for fasting log CORT, age bin, and BMI bin; *2*) for an effect of sex on each of EF (RHI), blood pressure, PP change in CRP, TNF-α, IL-1β, IL-6, IL-8, IL-10, and anger-induced change in HR, tonic skin conductance, and SCR, controlling for age bin, BMI bin, and fasting log CORT; and *3*) for an effect of sex on each of these dependent physiological variables, controlling for age bin, BMI bin, fasting log CORT, and average PP log CORT concentration. If the average PP log CORT concentration was significantly associated with these dependent variables evaluated in model “3,” and if there was a significant effect of sex on the average PP log CORT concentration under model “1,” we considered this to support PP log CORT as a mediator of the relationship between sex and cardiovascular, autonomic, inflammatory marker, and mood variables in question. To examine how inclusion of the average PP log CORT concentration variable (model 3) altered (mediated) the influence of sex on cardiovascular, autonomic, and mood variables, the adjusted means (least squares means) and the difference between the sex least squares means were compared between models without (model 2) and with (model 3) the PP cortisol variable in the model.

## Results

### Effects of sex on PP salivary cortisol

**FIGURE 2:**
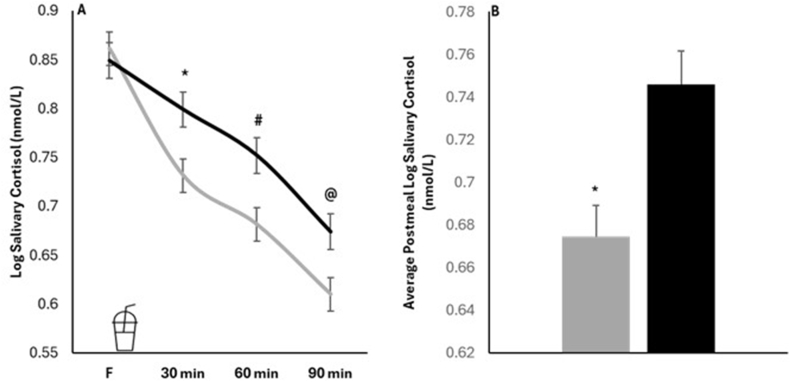
(A) A repeated measures mixed model yielded main effects of sex (*P* = 0.0190) and time (*P* = 0.0001), and a sex by time interaction (*P* = 0.0008), suggesting sex differences in the post meal patterns of salivary cortisol. A follow-up assessment using unadjusted t-tests at each timepoint showed differences between males and females at 30 min (∗*P* = 0.0068), 60 min (**#***P* = 0.0046), and 90 min (@*P* = 0.0103) after consumption of the test meal, but not during the pre-test meal (fasting) timepoint (*P* = 0.6200). Values represent adjusted means of the logarithm (log) of salivary cortisol concentrations. Gray: females (*n* = 183); black: males (*n* = 167). (B) Sex-related differences (∗*P* = 0.0009) in the average post-test meal salivary cortisol concentrations. Gray: females (*n* = 183); black: males (*n* = 167).

We found a significant sex by time interaction on PP cortisol (*P* = 0.0008) using a repeated measures mixed model. Females and males differed in the PP changes in log CORT, but not fasting log CORT; this sex by time interaction is displayed in Figure 2A. The average PP cortisol, statistically adjusted for pre-meal CORT, was also lower (*P* = 0.0009) in females (Figure 2B). Average log PP CORT concentrations in females were 0.67 ± 0.01 nmol/L, with a 95% confidence interval (CI, 0.64, 0.70 nmol/L). For males, log PP CORT concentrations were 0.74 ± 0.02 nmol/L, with a 95% CI (0.72, 0.78 nmol/L). We followed up to examine the association between sex and changes in log PP CORT at 30 min, 60 min, and 90 min PP. PP changes in log CORT, after adjusting for pre-meal values, were also significantly reduced in females (elevated in males) at 30 min (124% greater decrease in females, *P* = 0.0038), 60 min (72% greater decrease in females, *P* = 0.0019), and 90 min (37% greater decrease in females, *P* = 0.0030). For females, PP changes in log CORT were as follows: at 30 min (‒0.13 ± 0.02 nmol/L; 95% CI: ‒0.16, ‒0.10 nmol/L); at 60 min (‒0.18 ± 0.02 nmol/L; 95% CI: ‒0.21, ‒0.15 nmol/L); and at 90 min (‒0.25 ± 0.02 nmol/L; 95% CI: ‒0.28, ‒0.22 nmol/L). For males, PP changes in log CORT were as follows: at 30 min (‒0.06 ± 0.02 nmol/L; 95% CI: ‒0.09, ‒0.02 nmol/L); at 60 min (‒0.10 ± 0.02 nmol/L; 95% CI: ‒0.14, ‒0.07 nmol/L); and at 90 min (‒0.18 ± 0.02 nmol/L; (95% CI: ‒0.21, ‒0.15 nmol/L).

### Relationships between sex, PP cortisol, and anger-affiliated HR and SNS activity

**FIGURE 3:**
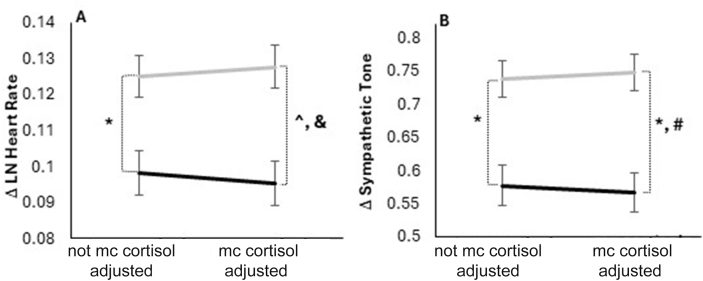
(A) The anger-recall task elevated heart rate in both females and males, with females having a greater increase in anger-induced heart rate during the anger task. Values represent adjusted means of the natural logarithm (ln) of heart rate (beats per minute). Controlling for post-test meal challenge (mc) salivary cortisol concentrations yielded an average 20% widening of anger-induced change (Δ) in heart rate between males and females. Although not graphically shown, higher post-test meal-cortisol concentrations were associated with elevated anger-induced increases in ln heart rate (β: 0.050 ± 0.022; *P* = 0.0232). ∗*P* = 0.0008; **ˆ***P* = 0.0001 (females, *n* = 165; males, *n* = 150). Gray: females; black: males. (B) The anger-recall task increased tonic skin conductance (sympathetic tone) in both females and males, with females having a greater increase in anger-induced sympathetic tone. Values represent adjusted means of the ln of tonic skin conductance in units of microSiemens (μS). #Controlling for post-test meal challenge (mc) salivary cortisol concentrations yielded a 12% widening of anger-induced change (Δ) in sympathetic tone between males and females. Although not graphically shown, higher post-test meal-cortisol concentrations were associated with elevated anger-induced increases in sympathetic tone (β: 0.23 ± 0.10; *P* = 0.0282). ∗*P* = 0.0001 (females, *n* = 165; males, *n* = 148). Gray: females; black: males.

Since both sex and glucocorticoids can influence cardiovascular and psychological function, we secondarily tested whether sex and average PP CORT concentrations influenced these functions later before and following the anger-recall task. Females had a greater increase in HR during the anger task (*P*_females compared with males_ = 0.002; Figure 3A) when average PP CORT was excluded from the statistical model. Change in natural logarithm (ln) HR for females was 0.1250 ± 0.0058 beats/min, with a 95% CI (0.1135, 0.1365 beats/min). For males, the change in ln HR was 0.0981 ± 0.0062 beats/min, with a 95% CI (0.0859, 0.1103 beats/min). However, after including the average PP CORT in the statistical model, we observed an average ∼20% widening of this sex-based difference in anger-affiliated HR elevation (*P*_females compared with males_ = 0.0003; Figure 3A). With PP CORT included, the change in ln HR for females was 0.1277 ± 0.0059 beats/min, with a 95% CI (0.1160, 0.1393 beats/min). For males, the change in ln HR was 0.0952 ± 0.0063 beats/min, with a 95% CI (0.0829, 0.1076 beats/min). Higher average PP CORT concentrations were associated with a greater increase in anger-induced HR (β: 0.050 ± 0.022; with a 95% CI for β: 0.007, 0.092; *P* = 0.0232). For every 1-unit increase in the log of cortisol, the natural log of HR increases by 0.05 units. Elevation in sympathetic tone (SNStone) during the anger task was also higher in females compared with males (*P* = 0.0001; Figure 3B). Change in ln SNStone for females was 0.7377 ± 0.0276 μS, with a 95% CI (0.6835, 0.7920 μS). For males, the change in ln SNStone was 0.5770 ± 0.0298 μS, with a 95% CI (0.5182, 0.6357 μS). However, this sex-based difference slightly widened by 12% when PP cortisol was included in the statistical model (Figure 3B). With PP cortisol included, the change in ln SNStone for females was 0.7469 ± 0.0277 μS, with a 95% CI (0.6924, 0.8014 μS). For males, the change in ln SNStone was 0.5665 ± 0.0300 μS, with a 95% CI (0.5074, 0.6256 μS). Higher average PP CORT concentrations are associated with a greater increase in anger-affiliated SNStone (β: 0.23 ± 0.10; with a 95% CI for β: 0.02, 0.44; *P* = 0.0282). For every 1-unit increase in the log of cortisol (nmol/L), the natural log of SNStone (μS) increases by 0.23 units.

### Effects of the anger task on CORT

Anger recall was associated with a decrease in log CORT in both males and females, and this decrease in CORT did not differ between the sexes at 30 min (*P* = 0.8832), 60 min (*P* = 0.5804), or 90 min (*P* = 0.7454) after initiation of the anger-recall task. In females, 30 min, 60 min, and 90 min log CORT changes were, respectively, (‒0.10 ± 0.01 nmol/L; 95% CI: ‒0.13, ‒0.07 nmol/L), (‒0.16 ± 0.02 nmol/L; 95% CI: ‒0.20, ‒0.13 nmol/L), and (‒0.14 ± 0.02 nmol/L; 95% CI: ‒0.18, ‒0.11 nmol/L). In males, 30 min, 60 min, and 90 min log CORT changes were, respectively, (‒0.10 ± 0.02 nmol/L; 95% CI: ‒0.13, ‒0.07 nmol/L), (‒0.15 ± 0.02 nmol/L; 95% CI: ‒0.18, ‒0.11 nmol/L), and (‒0.14 ± 0.02 nmol/L; 95% CI: ‒0.17, ‒0.10 nmol/L). Average PP CORT positively associated with anger-induced changes in CORT at 30 min (β: 0.22 ± 0.06; with a 95% CI for β: 0.11, 0.33; *P* = 0.0001) and 60 min (β: 0.21 ± 0.07; with a 95% CI for β: 0.08, 0.34; *P* = 0.0019), but the lack of differences between males and females in anger task-related changes in cortisol persisted after adding PP cortisol to the model. The average post anger-recall–related CORT concentrations did not associate with anger task-related increases in HR (β: 0.02 ± 0.02; with a 95% CI for β: ‒0.03, 0.06; *P* = 0.4721), SNStone (β: ‒0.08 ± 0.12; with a 95% CI for β: ‒0.30, 0.13; *P* = 0.4432), or anger (β: 0.10 ± 0.23; with a 95% CI for β: ‒0.35, 0.55; *P* = 0.6686).

To confirm that the anger task increased anger, participants filled out the POMS questionnaire before and after the anger task. With both sexes combined, the POMS questionnaire confirmed increases in anger (*P*_Δ__anger_ = 0.0001; 0.99 ± 0.04 rank change, with a 95% CI: 0.90, 1.08) immediately following the anger-recall task. Compared with males, females reported an 18% greater increase in anger after the recall task (*P* = 0.0489; females: 1.07 ± 0.06 rank change in anger, with a 95% CI: 0.96, 1.19; males: 0.91 ± 0.06 rank change in anger, with a 95% CI: 0.78, 1.02). PP cortisol did not associate with anger task-induced self-reported anger (β: 0.15 ± 0.22, with a 95% CI for β: ‒0.28, 0.58; *P* = 0.4903). The anger-recall task associated with increased frequency of SNS responses (SCR) in males (1.26 ± 0.05 ln mS; with a 95% CI: 1.16, 1.37 ln μS; *P* = 0.0001) and females (1.38 ± 0.05 ln mS; with a 95% CI: 1.28, 1.48 ln μS; *P* = 0.0001), with no differences between the sexes (*P* = 0.1031). PP CORT did not associate with anger-affiliated change in SCR [β: 0.23 ± 0.19, with a 95% CI: for β: ‒0.14, 0.60; *P* = 0.2224].

### Relationships between sex, PP CORT, and vascular function and blood pressure

**FIGURE 4:**
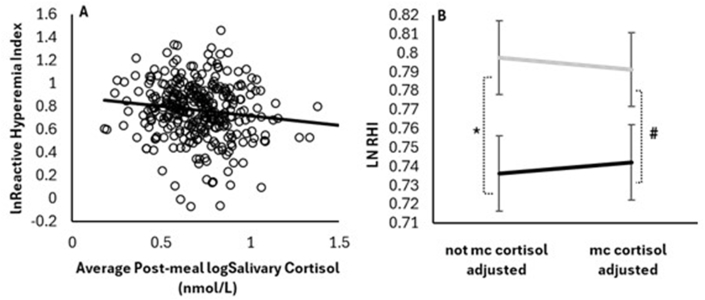
(A) Higher post meal salivary cortisol concentrations associated (*P* = 0.0197, *n* = 325) with a lower reactive hyperemia index (RHI) (lower endothelial function) independent of sex, BMI category, age category, and pre-meal salivary cortisol. Endothelial function was assessed at the end of the test visit, ∼8 h after the test meal. Natural logarithm (ln) RHI = ‒0.1637 × log cortisol + 0.8855; R^2^ = 0.02, *P* = 0.0197; *n* = 325. (B) Compared with females (*n* = 164), the RHI was lower (∗*P* = 0.0268) in males (*n* = 161). ^#^However, significantly controlling for differences in postprandial (mc) salivary cortisol concentrations reduced this sex-based difference in RHI by 20%, which was also no longer significant (*P* = 0.0807). Values represent adjusted means of the ln of the RHI and the logarithm of salivary cortisol concentrations. Gray: females; black: males.

Higher average PP CORT concentrations were significantly associated with lower EF (RHI) (β: ‒0.16 ± 0.07, with a 95% CI for β: ‒0.30, ‒0.03; *P* = 0.0197; Figure 4A). For every 1-unit increase in the log of cortisol, the natural log of RHI decreases by 0.16 units. The average anger task-affiliated CORT did not associate with EF (*P* = 0.4942). The RHI was lower in males without PP CORT in the model (*P* = 0.0268; Figure 4B). In females, the ln RHI was 0.7975 ± 0.0194, with a 95% CI (0.7594, 0.8357). For males, ln RHI was 0.7362 ± 0.0199, with a 95% CI (0.6971, 0.7753). However, with PP cortisol in the model, this sex-based difference in RHI did not reach significance (*P* = 0.0807; Figure 4B). Including PP cortisol in the model reduced the sex-based difference in RHI by an average of ∼20%. With cortisol included, the ln RHI in females was 0.7911 ± 0.0194, with a 95% CI (0.7528, 0.8293). For males, the ln RHI was 0.7422 ± 0.0199, with a 95% CI (0.7031, 0.7814). Blood pressure was also taken prior to conducting the EF procedure. At that time, males had significantly higher systolic blood pressure, with and without PP CORT included in the statistical models. Without cortisol in the model, systolic blood pressure was 118.0 ± 0.7 mmHg (95% CI: 116.6, 119.5 mmHg) in females and 122.4 ± 0.8 mmHg (95% CI: 120.9, 124.0 mmHg) in males (*P*_sex_ = 0.0001). With cortisol in the model, systolic blood pressure was 118.1 ± 0.7 mmHg (95% CI: 116.6, 119.6 mmHg) in females, and 122.4 ± 0.8 mmHg (95% CI: 121.0, 124.0 mmHg) in males (*P*_sex_ = 0.0001). Likewise, diastolic blood pressure was higher in males when PP cortisol was not included in the statistical model [(females, 66.9 ± 0.6 mmHg; 95% CI: 65.6, 68.1 mmHg) compared with (males, 71.3 ± 0.7 mmHg; 95% CI: 70.0, 72.6 mmHg), *P* = 0.0001] and was included [(females, 67.0 ± 0.6 mmHg; 95% CI: 65.7, 68.2 mmHg) compared with (males, 71.2 ± 0.7 mmHg; 95% CI: 69.9, 72.6), *P* = 0.0001]. At this time of day, PP cortisol did not associate with systolic [β: 2.06 ± 2.76, with a 95% CI: for β (‒3.36, 7.50); *P* = 0.4548] or diastolic (β: 1.96 ± 2.38, with a 95% CI: for β: ‒2.73, 6.65; *P* = 0.4112) blood pressure. Likewise, average post anger CORT did not associate with systolic (β: 1.84 ± 2.91, with a 95% CI for β: ‒3.88, 7.56; *P* = 0.5267) or diastolic (β: 1.24 ± 2.51, with a 95% CI for β: ‒3.71, 6.18; *P* = 0.6234) blood pressure.

### Relationships between sex, PP CORT, and inflammation markers

Refer to Table 1. PP changes in plasma concentrations of CRP, IL-1β, IL-8, and IL-10 did not differ between males and females and were not associated with meal-affiliated cortisol. Males and females differed (*P* = 0.0001) in the 3- and 6-h PP IL-6 response, with males showing a significant (*P* = 0.0001) decrease at 3 h and females having a significant (*P* = 0.0001) increase at 6 h PP. However, PP cortisol was not associated with IL-6 concentrations. PP changes in TNF-α concentrations did not differ between females and males, but PP cortisol was inversely associated with 6-h PP changes in TNF-α concentrations (*P* = 0.0028).

**TABLE 1:** Sex-related 3- and 6-h postprandial change in inflammatory markers

Variable^1^	Hour	Females			Males
		Mean ± SE^2^	95% CI	Mean ± SE	95% CI
CRP	3	‒0.015 ± 0.020	‒0.054, 0.024	‒0.008 ± 0.020	‒0.049, 0.032
	6	‒0.037 ± 0.019	‒0.075, 0.001	‒0.021 ± 0.020	‒0.061, 0.019
IL-1β	3	0.141 ± 0.034	0.073, 0.209	0.146 ± 0.036	0.073, 0.218
	6	0.102 ± 0.034	0.039, 0.164	0.106 ± 0.034	0.038, 0.173
IL-8	3	0.003 ± 0.001	0.001, 0.004	0.003 ± 0.001	0.001, 0.005
	6	0.002 ± 0.001	0.001, 0.003	0.002 ± 0.001	0.001, 0.004
IL-10	3	0.136 ± 0.020	0.095, 0.176	0.147 ± 0.021	0.104, 0.189
	6	0.096 ± 0.021	0.053, 0.138	0.051 ± 0.023	0.005, 0.097
TNFα^3^	3	‒0.192 ± 0.024	‒0.239, ‒0.145	‒0.177 ± 0.025	‒0.227, ‒0.127
	6	‒0.180 ± 0.024	‒0.228, ‒0.132	‒0.180 ± 0.026	‒0.231, ‒0.128
IL-6^4^	3	0.007 ± 0.007	‒0.007, 0.021	‒0.041 ± 0.007	‒0.056, ‒0.026
	6	0.052 ± 0.006	0.038, 0.066	‒0.001 ± 0.007	‒0.015, 0.013

Abbreviations: CI, confidence interval; CRP, C-reactive protein; IL, interleukin; ln, natural logarithm; SE, standard error; TNF-α, tumor necrosis factor α.

1 Data shown were derived from mixed models without meal-affiliated salivary cortisol in the model. Log (ln)-transformed data are shown for CRP (ng/mL), IL-1β (pg/mL), and IL-10 (pg/mL); square root-transformed data are shown for IL-8 (pg/mL) and IL-6 (pg/mL); and data for TNF-α (pg/mL) are presented in their original units.

2 All data shown represent adjusted values (least squares means) for 3- and 6-h change from baseline.

3 Although no differences in the TNF-α postprandial responses were observed between males and females, postprandial cortisol was inversely associated with 6-h postprandial changes in TNF-α concentrations (β: ‒0.27 ± 0.09, with CIs for β: ‒0.45, ‒0.09; *P* = 0.0028).

4 Males and females differed (*P* = 0.0001) in the 3- and 6-h postprandial IL-6 response.

## Discussion

Breakfast or the first meal of the day may reset activity in the HPA axis and reduce glucocorticoid concentrations (e.g., see [2]). If this resetting fails to occur, peak glucocorticoid concentrations persist for longer than normal over this period of the diurnal glucocorticoid rhythm. Compared with females who regularly ate breakfast, females who habitually skipped breakfast had elevated CORT concentrations throughout the morning and afternoon [2]. Results from that study also indicated a circadian-based decline in circulating cortisol concentrations from waking to the first meal of the day in both breakfast eaters and skippers. Together, a combination of circadian and dietary-based mechanisms likely influences the rate of cortisol decline after waking and following the first meal of the day. Expanding on these results in females, we asked whether cortisol concentrations that are affiliated with consuming the first meal of the day differed between males and females, and whether this possible sex-related variance in PP cortisol dynamics helps explain sex-related differences in cardiovascular and other glucocorticoid-sensitive functions later in the afternoon.

Although fasting CORT concentrations did not differ between females and males, our results indicate that females have a steeper reduction in systemic cortisol concentrations after consuming the first meal of the day. These findings, in combination with previous reports that implicate dietary-based contributions to the otherwise circadian-based cortisol decline after waking and following the first meal of the day, suggest that, after an overnight fast, meal consumption differentially influences the rate of systemic cortisol concentration decay in males and females. The observed difference in PP cortisol dynamics between males and females cannot be completely explained by age or BMI, since these variables were accounted for in our statistical models. It is well-established that meals, particularly the midday meal (lunch), stimulate increases in circulating cortisol concentrations (e.g., see [27,28]). This meal-induced cortisol response was shown to be higher in males [18,29], but much less is known about whether males and females differ in cortisol patterns after consuming the first meal of the day. Interestingly, in studies by Martens et al. [18] and Lemmens et al. [29] that showed sex-based differences in lunch-induced cortisol increases, fasted participants were provided a yogurt snack in the morning upon arrival to the laboratory in the fasted state. However, 2‒4 h after consuming the yogurt snack and before the experimental lunch meal, males were found to have higher CORT concentrations. Furthermore, in [29], fasting CORT concentrations examined prior to consumption of the yogurt snack did not differ between males and females. We can only speculate, but this might suggest that, like our findings, females had a higher rate of cortisol decline after consuming their first meal (yogurt) of the day. More studies are needed to determine whether and to what degree breakfast or the first meal of the day influences the circadian decrease in cortisol concentrations. Nevertheless, differences in the magnitude of this meal-affiliated cortisol decline may be particularly important since a diminished decline in cortisol concentrations after consuming the first meal of the day was shown in this study to associate with lower microvascular EF and elevated anger task-affiliated increases in HR and SNS tone.

Glucocorticoids such as cortisol play a significant role in cardiovascular function, and their alterations have long been affiliated with cardiovascular disease [30]. Even moderate elevations in cortisol concentrations or disruptions to the normal diurnal cortisol rhythm can be detrimental to several glucocorticoid-sensitive metabolic, cardiovascular, and behavioral functions [7,[31], [32], [33]]. Our findings are supportive of these prior reports and possibly suggest that sex-dependent variability in the cortisol response to breakfast explains, albeit modestly in this study, some cardiovascular and autonomic differences between males and females.

In support of our results showing an association between higher PP cortisol and lower reactive hyperemia, an elevated 30-min cortisol response to adrenocorticotropic hormone (ACTH) was reported to inversely associate with reactive hyperemia [34], and this relationship was not explained by differences in insulin sensitivity. Other studies have demonstrated vascular endothelial dysfunction in patients with Cushing’s syndrome, and impaired microvascular function and lower reactive hyperemia in persons treated with glucocorticoids [6,35,36]. Glucocorticoids can act via glucocorticoid or mineralocorticoid receptors in the vasculature to promote or enhance actions of the vasoconstrictors norepinephrine and angiotensin II [37,38], or of the SNS; each of these can alter vascular function [39,40]. Elevated SNS activity was reported to inversely associate with reactive hyperemia, as measured by the EndoPat method [41]. It is possible that higher PP cortisol augmented SNS reactions and/or heightened sensitivity to the effects of SNS outflow on vascular function. Glucocorticoids can also act via the hindbrain to promote enhanced norepinephrine and cardiovascular responses to psychological stress [42]. Including the average PP CORT concentration in the statistical model that tested for a sex difference in RHI reduced the difference in RHI between males and females by 20%. This suggests that the lower PP cortisol in females partially mediated the sex-related difference in this marker of EF.

Although we did not directly measure SNS activity or the SNS neurotransmitter norepinephrine, we did assess skin conductance activity before, during, and after the anger-recall task. We found that higher PP CORT was associated with greater increases in anger-recall–affiliated tonic skin conductance, but not SCRs. This suggests that higher PP cortisol may have partially contributed to anger-recall–affiliated increases in SNS tone [43]. SNS tone (tonic skin conductance) compared with rapid activation (SCRs) is regulated by different neural pathways, e.g., [43], and it is possible that a higher anger-recall–related change in SNS tone partially contributed to the association between higher PP cortisol concentrations, greater anger-affiliated changes in HR, and lower EF. Furthermore, studies implicate increased SNS tone in the mediation of exaggerated stress-affiliated SNS, cardiovascular, and glucocorticoid responses [44,45].

A prior study using a similar anger-recall procedure indicated that, compared with males, females may have greater HR and SNS responses to anger-recall [46]. Similarly, in our study, females had higher anger task-affiliated increases in HR and tonic skin conductance (SNS tone). However, these apparent effects of sex were further widened when significantly removing the sex differences in PP cortisol. Since PP cortisol was lower in females and positively associated with anger-affiliated increases in HR and tonic skin conductance, the observed widening effects of sex after controlling for differences in PP cortisol suggest that higher PP cortisol in males or lower PP cortisol in females canceled or dampened the effects of another pathway or pathways that mediate relatively greater anger-induced HR and skin conductance in females. These findings suggest that the lower PP cortisol concentrations in females may have mitigated further increases in anger-induced SNS tone and HR. It could also be the case that higher PP cortisol concentrations in males reduced the impact of pathways mediating lower anger-affiliated SNS tone and HR responses in males.

In that same study [46], males and females did not differ in their subjective responses to the anger-recall task. However, Engebretson et al. [47] showed females to respond to an anger-induction task with greater anger than males. Consistent with [47], our results suggest that, in this study, compared with males, females experienced heightened anger in response to the recall task. Elevated PP cortisol concentrations did not associate with anger after the recall task. Similar to our findings, anger induction has been shown to associate with decreases in cortisol [48,49], but others have found increases or no change in cortisol (see [48]). Since cortisol concentrations would typically continue to fall during the time we conducted the anger-recall task, and because we did not include a control task, we cannot say with certainty that the observed decreases in cortisol were due to anger induction. Nevertheless, anger-recall–affiliated decreases in cortisol concentrations did not differ between males and females.

It is also possible that the observed variability in post meal–affiliated cortisol patterns reflects broader differences in the overall circadian or ultradian rhythms of circulating cortisol concentrations. Less dynamic circadian (diurnal) and ultradian rhythms of cortisol and the HPA axis are linked to cardiovascular, autonomic, immunologic, and behavioral disturbances, as well as to altered stress responsiveness (e.g., [50,51]). Our examination of cortisol in this study was confined to the test visit. Therefore, we were unable to determine the extent to which circadian-related cortisol patterns differed between males and females in our study. Regardless of the mechanism, our results imply that sex-based differences in cortisol patterns or some factors related to cortisol regulation potentially contribute to autonomic and cardiovascular function differences in males and females. Since males and females differ in cardiovascular event and disease risk, and because anger and cortisol are linked to increases in cardiovascular events [52,53], our findings further support the need to take into consideration the relationships between sex, PP cortisol, and cardiovascular status in the context of anger. It is important to note that, based on some evidence [54], at least some of the physiological responses to the anger task may result from the narrative recall part of the anger task (compared with the feeling of anger). Therefore, future studies examining these relationships should include control tasks.

Lastly, our findings suggest a delay between PP cortisol in the morning and its implied effects on autonomic, cardiovascular, and mood status later during the test visit. This is not surprising given that glucocorticoids can have delayed and lasting effects. A study using either cortisol induction by infusing corticotropin-releasing hormone or infusion of hydrocortisone in humans to mimic the nocturnal rise in cortisol stimulated insulin resistance, and this effect developed 4–6 h after cortisol induction [55]. Moreover, this effect persisted for >16 h. The full effects of glucocorticoids on glycogen synthesis can also take hours to develop [56], and the report by Baque et al. [57] indicates a “priming” effect of glucocorticoids on the glycogenic capacity of hepatocytes that, when exposed to anabolic conditions, i.e., fed state and elevated insulin, undergo an increase in glycogen storage. Other researchers used hydrocortisone infusion and metyrapone-induced inhibition of cortisol synthesis to mimic the nocturnal cortisol elevation in persons with [58] and without [59] type 2 diabetes. In those studies, hydrocortisone infusion influenced carbohydrate metabolism after a mixed macronutrient meal, with lasting and full effects on some variables occurring hours after the glucocorticoid reached peak levels. Moreover, some neurological effects of glucocorticoid induction appear to be lasting and require hours to develop [[60], [61], [62]]. Together, it can take hours for glucocorticoids to exert their full effects on some physiological, metabolic, and neurological functions. Therefore, in this study, it is not terribly surprising to have observed a relationship between PP cortisol concentrations in the morning and cardiovascular and autonomic function later in the afternoon. However, in this observational study, it’s not possible to determine whether this association reflects direct or indirect effects of PP cortisol or an independent factor common to PP cortisol regulation and the regulation of these other functions. Nevertheless, our results suggest that variation in the cortisol patterns after consuming the first meal of the day may partially explain sex-related differences in and independently influence cardiovascular and SNS status later during the day.

We acknowledge that there are limitations associated with this study. The study used a mixed macronutrient challenge test meal high in saturated fat and with sucrose as the sole carbohydrate source. It is, therefore, important to note that meals of different forms and compositions may elicit different responses and outcomes. Since this was an observational study, we cannot assert mechanisms of action or causation from our findings. However, our results are consistent with multiple studies supporting nutritional regulation of the HPA axis and circulating glucocorticoid concentrations. We also acknowledge that, at least in this study population, the amount of variation explained in our association results is relatively small. However, these results do provide further support for sex-based differences in cortisol and this hormone’s potential to partially explain autonomic and cardiovascular differences in females and males. Finally, because this is a secondary analysis of the “Nutritional Phenotyping Study” [19], the sample size was not calculated based on this particular analysis. As we were able to find significant associations, we believe the sample size was adequate.

In conclusion, overall, our findings distinctly imply that variability in cortisol concentration patterns after consuming the first meal of the day partly contributes to sex-related differences in autonomic and cardiovascular function later in the day and possibly in response to emotion-induction. Relative to males in this study, a steeper decline in cortisol after eating the first meal of the day may partly help mitigate the magnitude of elevated SNStone and increases in HR, whereas facilitating EF in females later during the day and/or in response to emotion-induction. Furthermore, it is also feasible that higher PP cortisol concentrations in males reduced the impact of pathways mediating lower anger-affiliated SNS tone and HR responses in males. More studies are needed to confirm our results and to test for a possible physiological connection between breakfast-related cortisol regulation, cardiovascular status, and reactions to anger and other emotions. Since inflexible cortisol and metabolic responses to meals are linked to poor health, a deeper understanding of whether and how these neuroendocrine and metabolic variables mechanistically interact could partially help explain differences in cardiovascular resilience or vulnerability.

## Author contributions

The authors’ responsibilities were as follows– KDL, NLK: contributed to the primary study design; KDL, RGS, NLK: contributed to the overall conceptual development of the current study; RGS: advised on the analysis of inflammation markers; KDL: wrote the manuscript and conducted data analysis with advice from the Western Human Nutrition Research Center consulting statistician, Jan Peerson; KDL: submitted the manuscript; all authors contributed equally to editing the manuscript; and all authors: read and approved the final manuscript.

## Data availability

Data described in the manuscript will be made available upon request, pending application and approval.

## Funding

This research was supported with funding from USDA-Agricultural Research Service projects 2032-51530-022-00-D and 2032-51530-025-00-D.

## Conflict of interest

The authors report no conflicts of interest.
